# Self‐reported sensitivity to pain in early and moderately‐late preterm‐born adolescents: A community‐based cohort study

**DOI:** 10.1002/pne2.12053

**Published:** 2021-05-11

**Authors:** Nienke H. van Dokkum, Marlou L. A. de Kroon, Sijmen A. Reijneveld, Arend F. Bos

**Affiliations:** ^1^ Department of Pediatrics Division of Neonatology Beatrix Children’s Hospital, University Medical Center Groningen, University of Groningen Groningen the Netherlands; ^2^ Department of Health Sciences University Medical Center Groningen, University of Groningen Groningen the Netherlands

**Keywords:** adolescence, inotropic agents, pain sensitivity, pain syndromes, prematurity

## Abstract

We aimed to compare ratings of self‐reported and parent‐reported pain sensitivity between early preterm (EP), moderately‐late preterm (MLP), and full‐term (FT) adolescents. For EP adolescents, we aimed to determine whether pain sensitivity was associated with early‐life events. EP (n = 68, response rate 47.4%), MLP (n = 128, response rate 33.0%), and FT (n = 78, response rate 31.1%) adolescents and their parents (n = 277) answered an author‐generated question on pain sensitivity at 14‐15 years of age within a community‐based cohort study. Differences between groups were determined using the chi‐square test for trends. For EP adolescents, we assessed associations of treatment modalities (inotrope treatment, mechanical ventilation, and C‐section) and neonatal morbidities (sepsis/necrotizing enterocolitis, small‐for‐gestational age status, asphyxia, and cerebral pathologies) with adolescent pain sensitivity using logistic regression analyses. Increased pain sensitivity was reported by 18% of EP adolescents, compared with 12% of MLP adolescents, and 7% of FT adolescents (*P* = 0.033). Parent‐reported pain sensitivity did not differ by gestational age group. For EP adolescents, inotrope treatment was associated with increased pain sensitivity (odds ratio, 5.00, 95% confidence interval, 1.23‐20.4, *P* = 0.025). No other neonatal treatment modalities or morbidities were associated with pain sensitivity in adolescence. In conclusion, we observed higher proportions of increased pain sensitivity for EP and MLP adolescents. Physicians treating preterm adolescents should be aware of altered pain sensitivity.

## 1 INTRODUCTION

Preterm birth occurs in approximately every one in ten pregnancies.^1^ Most of these children are born moderately‐late preterm (MLP, ie, between 32 and 37 weeks’ gestation) and the remainder are born early preterm (EP, that is before 32 weeks’ gestation). Especially for EP infants, their start in life can be difficult and involves essential treatment in a neonatal intensive care unit (NICU). During their NICU stay, neonates experience physical and sensorial events, including painful procedures and mechanical ventilation, excess of noise and light, and maternal separation.^2^ This all occurs at a time when the developing brain is extremely sensitive.^3^, ^4^ It has been firmly established that these neonates are at high risk of a variety of problems later in life, including adolescence, such as impaired growth,^5^ developmental delay,^6^ and behavioral problems.^7^

One specific area of problems that becomes manifest later in life concerns pain‐processing, with evidence being limited. Pain and sensitivity to pain have a complex nature. The International Association for the Study of Pain has defined pain as “An unpleasant sensory and emotional experience associated with, or resembling that associated with, actual or potential tissue damage.”.^8^ Pain and pain sensitivity are also influenced by biological factors such as early life experiences,^8^, ^9^ psychological factors,^8^ and social factors such as educational level.^8^, ^10^ It is hypothesized that preterm birth and painful sensory experiences during the neonatal period result in changes of an individual's pain‐processing system, both in the short and in the long term.^11^, ^12^, ^13^ We found only a few studies that investigated this hypothesis using formal assessments. One of these reported increased tenderness of muscle trigger points in preterm adolescents.^13^ Another reported lower pain tolerance in preterm adolescents as measured by the Cold Pressor Task, which records the number of seconds in cold water until withdrawal.^10^ A third study reported greater pain sensitivity in NICU graduates at school‐age as measured by thermal and mechanical stimulation.^14^ Recently, another study reported similar thermal detection rates and pain sensitivity for very‐low‐birthweight adults compared with full‐term (FT) controls.^15^ Studies are also limited regarding specific early life experiences that may be associated with altered pain sensitivity. The few available studies report that neonatal surgery, necrotizing enterocolitis, mechanical ventilation, and sex were associated with pain responses later in life.^15^, ^16^, ^17^

There is even less evidence regarding self‐reported pain sensitivity, particularly in adolescence, for EP and MLP children. In line with the findings on formal assessments, self‐reported pain sensitivity may be higher in preterm‐born adolescents. Increased self‐reported pain sensitivity is an important characteristic because higher sensitivity to pain may increase the perceived procedure‐related intensity of pain and may increase the risk of complex regional pain syndromes or fibromyalgia.^18^ Literature on daily health complaints and pain‐related symptoms reports conflicting findings. Some studies report that preterm adolescents did not have daily health complaints or pain‐related symptoms more frequently than FT peers,^10^, ^12^, ^13^, ^19^ while others report a difference.^11^ Moreover, some studies also report associations between pain‐related symptoms, for example migraines or leg cramps, and early life factors, such as NICU admittance or neonatal surgery.^20^, ^21^ Rates of daily health complaints or pain‐related symptoms may be similar between preterm and FT adolescents, but even then hypersensitivity to pain could still increase procedure‐related pain intensity. If procedure‐related pain intensity is indeed increased, then we may need long‐term follow‐up programs in preterm children to monitor and facilitate how they cope with higher pain sensitivity and associated syndromes, such as fibromyalgia and chronic pain. In addition, identifying sensory experiences associated with sensitivity to pain may help to select those children who would benefit most from such a follow‐up program in the future.

Moreover, in adolescence, there may be considerable variability in the extent to which children have control over their medical follow‐up and treatments, and parents may still have an important role. Studies have also reported that parents may shape their children's responses to pain.^10^, ^22^, ^23^ For preterm children especially, parents have been involved since birth and admittance to the NICU, which may increase the risk of being more concerned about pain experiences, even in adolescence. It is unknown to what extent parent‐reported and self‐reported pain sensitivities correspond at this specific age.

Therefore, we aimed to compare the ratings of self‐reported and parent‐reported sensitivity to pain of EP, MLP, and FT adolescents and, in EP adolescents, to determine whether sensitivity to pain was associated with early‐life events.

## 2 METHODS

### 2.1 Setting and population

**FIGURE 1:**
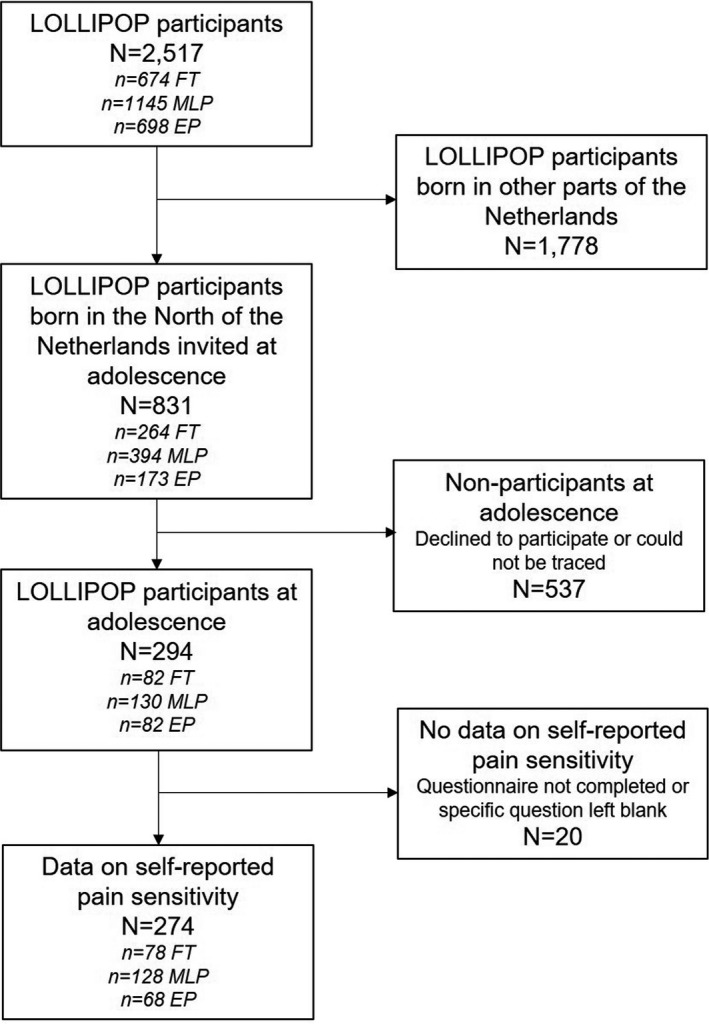
Flow of participants. EP, early preterm; FT, full‐term; MLP, moderately‐late preterm

This study was part of the Longitudinal Preterm Outcome Project (LOLLIPOP).^6^ This project comprised children born between 2002 and 2003 who were included at the time of their last visit to their well‐child center at 43‐49 months. Upon inclusion, 25% of a national year cohort was screened for eligibility. Every preterm‐born child with a gestational age of <36 weeks was included. After every two preterm children, a FT child from the same source was included as a control. Children with major congenital malformations and syndromes were excluded. For the present study, we invited all children living in the three northern provinces of the Netherlands to participate in the follow‐up wave at adolescence. A total of 294 children (82 EP, 130 MLP, and 82 FT) participated between April 2017 and November 2018. The response rates were 47.4% (173 invited), 33.0% (394 invited), and 31.1% (264 invited) for the three gestational age groups, respectively. Other children either declined or could not be traced. The flow of participants is depicted in Figure 1. Prior to the start of the follow‐up wave, a power analysis was performed for the primary outcome measure (ie, intelligence quotient), on which the required participants were calculated with effect size 0.33, using a power of 80%. With about 80% power, we were also able to detect differences in prevalences of dichotomized outcomes between groups of around 0.25 versus 0.10. The eventually included number of participants was slightly higher than aimed for in the EP and FT group (required number 71 participants) and slightly lower in the MLP group (required number 142 participants). All parents provided written informed consent at the start of LOLLIPOP. Both parents and children provided written informed consent to participate in the follow‐up wave at adolescence. LOLLIPOP, including the follow‐up wave at adolescence, was approved by the Medical Ethical Review Board of the University Medical Center Groningen (METc 2005/130 and METc 2017/01), the Netherlands.

### 2.2 Self‐reported and parent‐reported pain sensitivity

As part of the follow‐up wave in adolescence, participants were asked to complete several questionnaires. One of the questionnaires focused on health behaviors and included one question on self‐reported sensitivity to pain. This question read: “How do you experience pain compared with your peers?” and the choice was between the following answers: (a) less sensitive, (b) equally sensitive, and (c) more sensitive. Parents were also asked to complete a general questionnaire that included a question on their children's sensitivity to pain. This question was: “How, in your opinion, does your child experience pain compared with his or her peers?” and they could choose one of the following answers: (a) less sensitive, (b) equally sensitive, and (c) more sensitive.

### 2.3 Participant characteristics, neonatal treatment modalities, and neonatal morbidities

The characteristics of the participants were collected using general questionnaires when LOLLIPOP started, a medical chart review, and a parental questionnaire. Perinatal characteristics included gestational age, sex, and small‐for‐gestational age status. Gestational age was verified by early ultrasound measures in over 95% of the cases. Retrospectively, early‐life events, including neonatal treatment modalities (inotrope treatment, mechanical ventilation, and C‐section) and neonatal morbidities (sepsis/necrotizing enterocolitis, small‐for‐gestational age status, asphyxia, and cerebral pathologies), were collected by reviewing the infants’ medical charts. These factors were included because of their association with neurodevelopmental impairments. Pain processing has been suggested to be an integrated part of neurodevelopmental functioning.^10^ Therefore, the aforementioned factors may also be associated with sensitivity to pain. Seeing that all EP infants were admitted to a single NICU, data registrations and treatment indications were consistent. Small‐for‐gestational age status was determined as a birthweight below the 10th percentile on the Dutch Kloosterman curves.^24^ Asphyxia was defined as an Apgar score of <7 at 5 minutes of age. Cerebral pathologies were assessed using serial cranial ultrasound measurements and classified as existing when either a grade 3 or grade 4 bleed and/or cystic periventricular leukomalacia was present. These early‐life events were dichotomized (present versus absent, or yes versus no).

### 2.4 Statistical analysis

We first described the participant characteristics and perinatal characteristics of the included adolescents. Next, we compared the ratings of self‐reported and parent‐reported sensitivity to pain in three categories (more sensitive, equally sensitive, and less sensitive) of EP, MLP, and FT adolescents using the chi‐square test for trends, also known as the Cochran‐Armitage test. We repeated the self‐reported sensitivity to pain analyses stratified by sex. We also described agreement between self‐reported and parent‐reported sensitivity to pain responses and tested it using Cohen's kappa. Because the trend toward increased sensitivity to pain appeared for preterm adolescents, specifically for EP adolescents, we assessed whether several neonatal morbidities and treatment modalities were associated with increased self‐reported sensitivity to pain in adolescence for the EP group, using logistic regression analyses with a dichotomized outcome measure (more sensitive versus the other two categories). Sex and maternal educational level were considered potential confounding factors and were added to the model to adjust when they were associated with both sensitivity to pain as outcome and the determinant early life factor (*P* < 0.1 using chi‐square tests). All analyses were performed using SPSS, version 26.0 (IBM, Chicago, Illinois, USA). The *P* values below 0.05 were considered statistically significant.

## 3 RESULTS

### 3.1 Participant characteristics

Altogether 274 adolescents (93%) reported on sensitivity to pain, 78 of whom were born FT, 128 were born MLP, and 68 were born EP. Of these EP adolescents, 13 (19%) were born extremely preterm (ie, with a gestational age below 28 weeks). For the remaining 20 children, questionnaires were either missing or this specific question had been omitted. Parents of 277 adolescents (94%) completed the question on sensitivity to pain. For the remaining 17 children, most parents did not accompany their adolescent child to the follow‐up appointment and had therefore not completed the questionnaires. Participant characteristics are presented in Table 1. Almost half of the adolescents (47%) were boys, distributed equally across the three gestational age groups. A total of 46 children (16%) were born small‐for‐gestational age. Characteristics differed between participants and nonparticipants (Table S1), with relatively more females, children born EP and small‐for‐gestational age, and children from higher socioeconomic status participating.

**TABLE 1:** Participant characteristics of early preterm, moderately‐late preterm, and full‐term adolescents

Characteristic	Full‐term n = 78	Moderately​‐late preterm n = 128	Early preterm n = 68	*P*‐value
Age at follow‐up (years)	15.4 (15.0‐15.8)	15.8 (15.3‐16.1)	14.9 (14.2‐15.5)	**<0.001**
Gestational age (weeks)	40 (39‐40)	34 (33‐35)	29 (28‐30)	**<0.001**
Birthweight (grams)	3520 (3210‐3860)	2220 (1830‐2550)	1225 (985‐1626)	**<0.001**
Small‐for‐gestational age	8 (9.6)	20 (15.3)	18 (22.5)	0.077
Sex				
Male	38 (46.3)	64 (48.9)	36 (45.0)	0.85
Female	44 (53.7)	67 (51.1)	44 (55.0)	
Maternal educational level				
Low/middle	52 (63.4)	77 (61.6)	58 (72.5)	0.26
High	30 (36.6)	48 (38.4)	22 (27.5)	

Note

Maternal educational level was measured upon inclusion in the LOLLIPOP study (age 4 years) and categorized as: low/middle educational level, that is, <12 years of formal education and high educational level, that is, ≥12 years of formal education. Data are reported as median (interquartile range) or n and (percentages [%]) where appropriate. Differences were tested with Kruskal‐Wallis tests for continuous variables and chi‐square tests for dichotomous variables.

Bold printed ​*P* values indicate statistically significant values <0.05.

### 3.2 Self‐reported and parent‐reported pain sensitivity at adolescence

**FIGURE 2:**
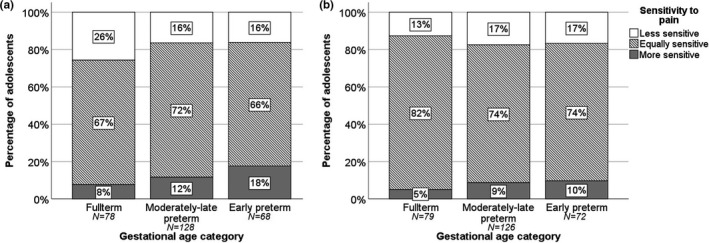
Self‐reported (A) and parent‐reported (B) sensitivity to pain of early and moderately‐late preterm adolescents compared with full‐term adolescents. Chi‐square test for trends, *P* = 0.033 and *P* = 0.94, respectively

**FIGURE 3:**
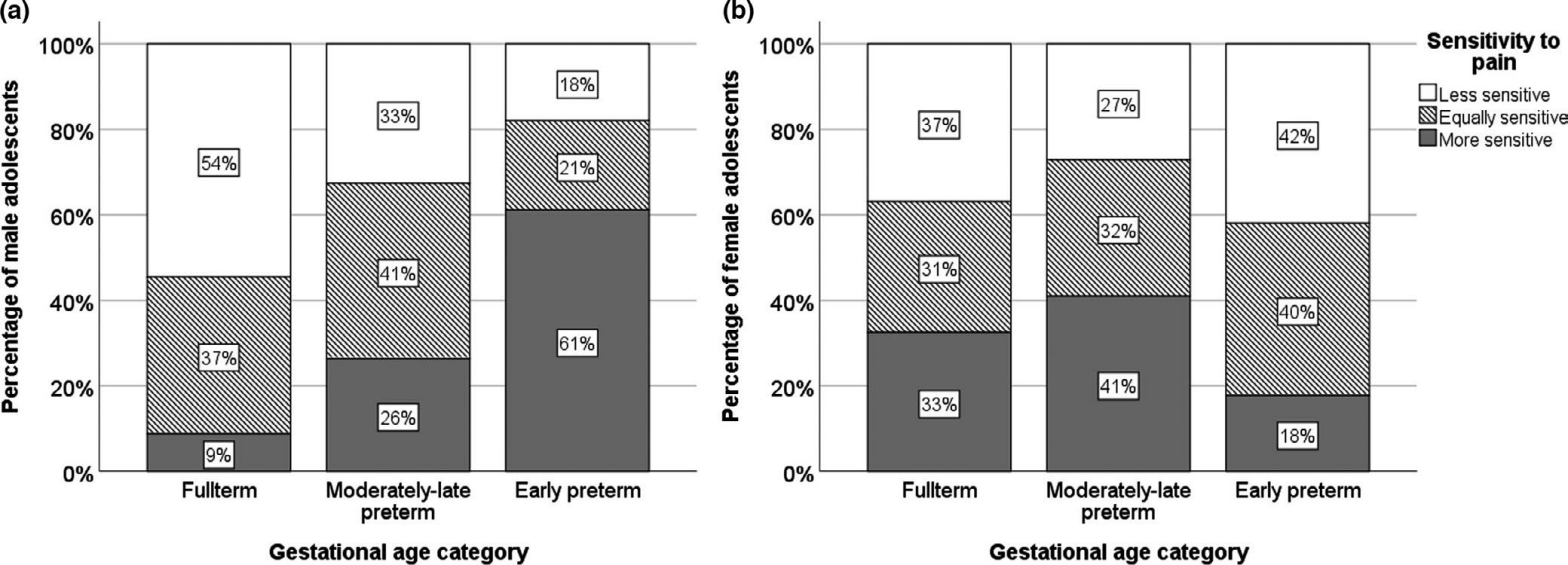
Self‐reported sensitivity to pain in male (A) and female (B) full‐term, moderately‐late preterm. and early preterm adolescents. Chi‐square test for trends, *P* = 0.002 for boys and *P* = 0.64 for girls

Most of the adolescents stated that they were equally sensitive to pain as their peers. Nevertheless, 18% of the EP adolescents reported to be more sensitive to pain than their peers, compared with 12% of the MLP adolescents and 7% of the FT adolescents (chi‐square test for trends, *P* = 0.033). Moreover, 16% of the EP adolescents experienced pain less sensitively, compared with 17% of MLP adolescents, and 26% of FT adolescents (Figure 2). Most of the parents (79%) reported their adolescent child as being equally sensitive to pain. No statistical differences were found regarding the distribution of increased sensitivity to pain reported by parents between gestational age groups (*P*= 0.94; Figure 2). Stratified by sex, we found this trend to hold for boys, but not for girls (Figure 3). For boys, we found the highest percentage of reported sensitivity to pain in EP adolescents, whereas for girls, this occurred in the MLP adolescents. In 259 cases (88%), both the adolescents and the parents completed the sensitivity to pain question. We present the agreement between these responses for each gestational age category in Table 2. Overall, 68% of responses were in agreement, with a kappa of 0.344 (*P* < 0.001). In total, 17% of the adolescents considered themselves to be more sensitive to pain than their parent score indicated and 15% considered themselves less sensitive to pain than their parents reported them to be.

**TABLE 2:** Agreement between self‐reported and parent‐reported ratings on sensitivity to pain for early preterm, moderately‐late preterm, and full‐term adolescents

Category	Full‐term n = 75	Moderately‐late preterm n = 122	Early preterm n = 62	Total N = 259
In agreement, n (%)	53 (70.7)	83 (68.0)	41 (66.1)	177 (68.3)
Child scores more sensitive than parent, n (%)	8 (10.7)	22 (18.0)	13 (21.0)	43 (16.6)
Child scores less sensitive than parent, n (%)	14 (18.7)	17 (13.9)	8 (12.9)	39 (15.1)

Note

Data are reported as n and (percentages [%]). Because of rounding, percentages do not always add up to 100%.

### 3.3 Early‐life events associated with pain sensitivity in early preterm adolescents

In Table 3, we present the associations of several early‐life events (neonatal treatment modalities and neonatal morbidities) with increased sensitivity to pain in EP adolescents. Of these, treatment early in life with inotropes was the only event that was associated with increased self‐reported sensitivity to pain. The odds ratio was 5.00 (95% confidence interval, 1.23‐20.4, *P* = 0.025). Because neither sex or maternal educational level were associated with both sensitivity to pain and treatment with inotropes, these factors were not added to the model.

**TABLE 3:** Associations of early‐life events associated with increased versus not increased self‐reported sensitivity to pain in early preterm adolescents: results of logistic regression analyses

Early life event	n present/n total (% present)	OR	95% CI	*P* value
Treatment modalities				
Delivery by C‐section	37/67 (55.2)	0.97	0.26‐3.55	0.96
Mechanical ventilation	44/67 (65.7)	2.70	0.53‐13.7	0.23
Inotropes	13/67 (19.4)	5.00	1.23‐20.4	**0.025**
Neonatal morbidities				
Asphyxia	7/65 (10.8)	2.18	0.37‐13.0	0.39
Small‐for‐gestational age	16/68 (23.5)	0.60	0.12‐3.08	0.54
Sepsis/necrotizing enterocolitis	20/65 (30.8)	2.67	0.68‐10.5	0.16
Cerebral bleeding grade 3 or 4, or cystic PVL	8/61 (13.1)	0.61	0.07‐5.57	0.67

Note

Asphyxia was defined as an Apgar score <7 at 5 min. Small‐for‐gestational age was defined as a birthweight for gestational age below the 10th percentile on the Dutch Kloosterman curves.

Abbreviations: CI, confidence interval; OR, odds ratio; PVL, periventricular leukomalacia.

Bold printed ​*P* values indicate statistically significant values <0.05.

## 4 DISCUSSION

In this study, we aimed to compare ratings of self‐reported and parent‐reported sensitivity to pain of EP adolescents, MLP adolescents, and FT adolescents. For EP adolescents, we also aimed to determine whether sensitivity to pain was associated with early‐life events. We demonstrated that approximately one in five EP adolescents reported increased sensitivity to pain compared with one in fourteen for their FT peers. This higher sensitivity to pain was associated with inotrope treatment during their stay in the NICU. The proportion of MLP adolescents reporting increased sensitivity to pain was in between those of EP and FT adolescents, approximately one in eight children. The proportion of EP and MLP adolescent reporting lower sensitivity to pain was less than that of FT adolescents. Parent‐reported pain sensitivities did not differ between the gestational age groups.

Our most important finding was the higher prevalence of increased sensitivity to pain associated with preterm birth, especially in EP adolescents. This is in line with the literature, however scarce, on pain sensitivity experiments in adolescents^10^, ^17^ and the increased self‐reported pain sensitivity in adolescents, with lower pain thresholds and more tenderness points for EP adolescents compared with FT peers.^13^ Our study added to these findings using a large sample of children at a particularly important moment during follow‐up. It is striking that even 14 to 15 years after a prolonged NICU stay, the rate of self‐reported increased sensitivity to pain of EP adolescents is still 2.5 times higher than that of FT adolescents. In line with our findings, Oberlander and colleagues reported that at four months’ corrected age, EP infants expressed greater facial pain response to finger lances after discharge from the NICU compared with FT infants.^25^ In contrast with our findings, Iversen and colleagues reported similar thermal detection and pain sensitivity in very‐low‐birthweight adults compared with FT controls.^15^

An explanation of this altered sensitivity to pain in EP children may be central sensitization, reflecting an abnormal state of responsiveness of the nociceptive system, which is responsible for pain responses.^26^ With prolonged pain experiences by noxious stimuli in early postnatal life, nociception is altered through enhancements of neuronal circuits in the central nervous system, resulting in altered pain thresholds and hypersensitivity to painful stimuli.^26^, ^27^ The postnatal environment during NICU stay, including the painful experiences that EP infants undergo, has been associated with reduced white matter volumes and altered microstructure development of the brain, which underlines this central sensitization hypothesis.^28^

Strikingly, we found this higher prevalence of increased sensitivity to pain to hold true only for boys, with EP adolescents reporting more sensitive pain experiences most often. In contrast, for girls, we found the MLP adolescents reporting more sensitive pain experiences most often. This finding contradicts those of other studies in EP children that reported males to be more resilient to pain than females.^13^, ^15^, ^17^ An explanation may be that sex‐dependent differences are influenced by tissue injury and pain in early life, contributing to activity‐dependent alterations in nociceptive signaling.^16^ We speculate that boys have a higher illness severity during NICU stay and are therefore exposed to more skin‐breaking procedures, contributing to these altered pain‐processing circuits. Regarding the relatively high percentage of female MLP adolescents reporting being more sensitive to pain, psychological factors of resilience may play a role. These sex‐dependent differences in sensitivity warrant further elucidation.

Furthermore, we found treatment with inotropic agents during NICU stay to be associated with the increased sensitivity to pain in EP adolescents. As far as we are aware, this is a new finding; we have not found other studies that link inotrope treatment during NICU stay to increased pain sensitivity. This finding might be the result of chance due to multiple comparisons, but there may also be a true association. Two pathophysiological mechanisms may explain the increased sensitivity to pain. First, the inotropic agents may directly affect pain processing in the brain. Dobutamine and dopamine were the two main inotropic agents used in our NICU at the time these children were treated there. Nevertheless, because dopamine and dobutamine do not pass the blood‐brain barrier, we expect these direct effects to be limited. A second and more likely explanation may be that inotrope treatment with these two agents is an expression of severe circulatory insufficiency, because it is a second‐line treatment modality, after fluid boluses. Circulatory insufficiency may be a complication of several severe morbidities, such as asphyxia, necrotizing enterocolitis, sepsis, and large intraventricular hemorrhages, indicating that the infants are severely ill. Inotrope treatment may therefore reflect impaired oxygenation of the brain as well as the many painful skin‐breaking procedures required to monitor these children, whatever the underlying cause of the severe circulatory insufficiency. This explanation is supported by the associations of inotrope treatment with developmental delay in preterm children at the age of two years^29^ and of lower cerebral oxygenation with impaired neurodevelopment.^30^ Still, these factors (ie, circulatory insufficiency, inotrope treatment, skin‐breaking invasive procedures, and illness severity) may be highly correlated, and it is therefore difficult to entangle whether it is inotrope treatment in itself or the associated exposure to early life pain that forms the basis of the identified association. Future studies should try to disentangle these factors and investigate whether treatment with inotropic agents is associated with altered cerebral oxygenation, to further elucidate the pathophysiology of altered pain sensitivity in EP adolescents.

We were surprised that none of the other investigated neonatal morbidities and treatment modalities were associated with changes in pain sensitivity in EP adolescents. In contrast to our findings, previous research showed associations of other neonatal treatment modalities, such as necrotizing enterocolitis and neonatal surgery, with altered pain sensitivity.^19^, ^31^ We speculate that this contrast may be due to the increased occurrence of skin‐breaking procedures associated with these modalities. In our study, only a few infants suffered from necrotizing enterocolitis, which limited the power to find associations. The remaining EP children may vary in the number of skin‐breaking and pain‐related procedures that they experienced due to varying illness severity and this heterogeneity may have obscured associations with higher pain sensitivity in adolescence in the full group of EP. A second explanation could be limited statistical power to detect associations between these neonatal morbidities and treatment modalities and sensitivity to pain in EP adolescents at all.

Another important finding was that MLP adolescents reported higher sensitivities to pain more frequently than FT peers, but less frequently than EP peers. To the best of our knowledge, ours is the first study that included MLP adolescents in a pain sensitivity study. Our findings align with the central sensitization hypothesis, because MLP infants are often admitted to a NICU for a shorter period of time or not at all. The early postnatal environment, including painful experiences, thus resembles to that of FT peers. Thus, even though brain maturation is not fully completed,^4^ and MLP infants are at risk of a variety of problems later in life,^32^ increased sensitivity to pain seems to be less of problem than it is for EP children.

Finally, parent‐reported sensitivity to pain did not differ between the gestational age groups, even though in 68% of the cases responses of the adolescents and parents were in agreement. In contrast to our findings, parents of extremely low birth weight infants did report that their children were less sensitive to pain from bumps, scrapes, and falls at school‐age.^33^ This contrast may be due to a different conceptualization of pain. Parents may consider responses to bumps, scrapes, and falls differently than responses to procedural pain. Our findings align with previous studies suggesting discrepancies between parent‐reported and self‐reported pain sensitivities in adolescence.^34^, ^35^ For clinical practice, it is therefore important to allow the self‐reports of preterm adolescents to prevail over parental reports.

### 4.1 Strengths and limitations

The strength of this study is the longitudinal character of LOLLIPOP, in which a large community‐based sample of both EP and MLP infants were followed up until adolescence. Moreover, we could investigate a broad range of neonatal treatment modalities and neonatal morbidities in EP adolescents. A limitation of this study is that sensitivity to pain was assessed by means of an author‐generated questionnaire and not by a validated questionnaire, nor cross‐checked with formal assessment data because we did not perform experiments. As all three gestational age groups completed the same question, we believe that the potential bias associated with this question would be similar across the groups and therefore would not affect differences as found. Still, we believe that self‐reported sensitivity to pain, a phenomenon that is difficult to quantify, is important for follow‐up programs, and more easily obtainable than data from physical assessments. Another limitation of this study is that our medical chart review did not encompass neonatal skin‐breaking procedures, pain‐related procedures or morphine exposure, because this chart review was retrospective and neonatal nurses were, at that time, not instructed or trained to record these data. Inclusion of neonatal pain data may have strengthened our results, because the number of skin‐breaking procedures has previously been a strong predictor of adverse cognitive and developmental outcomes.^28^ Moreover, our study did not include psychological factors such as anxiety and depression, because these data were not available in our community‐based cohort. These factors might have influenced the results of this study and could have clarified the identified associations slightly more. We also did not investigate pain coping of the adolescents. Our focus on this follow‐up wave at adolescence was quite broad, investigating health, health‐related behavior, and neuropsychological development of the adolescents. The participants were asked to complete several extensive questionnaires, but data on pain coping were unfortunately not available in our cohort. We were also unable to incorporate social learning as a concept in this study, even though children's understanding of pain and how to express pain may affect pain experiences and coping.^8^ It may well be, for example, that the degree of prematurity had influenced the way parents coped with their children's pain differently between boys and girls. Finally, the response rate in our study was relatively low and the participatory sample included slightly more females, children born EP, children born small‐for‐gestational age, and children from high socioeconomic status. Although effect sizes were small, this might have introduced some bias. Moreover, we had a relatively low number of adolescents in our EP subsample. Studies with samples sizes below 100 may overestimate the effect measure, so our findings have to be confirmed in further large‐scale studies.

### 4.2 Implications

Our findings imply that follow‐up programs targeting preterm‐born children should incorporate sensitivity to pain. Because these children also seem to have altered coping styles, displaying greater catastrophizing of painful events,^22^, ^36^ we speculate they may have increased risks of associated pain syndromes, although we did not study this. We believe it makes sense to incorporate coping with pain as well as sensitivity to pain in follow‐up programs, during both childhood and adolescence. Physicians treating preterm children or adolescents should be aware that during painful procedures they may display increased sensitivity to pain and may possibly have associated pain syndromes. Future studies should focus on the interplay between sensitivity to pain, pain syndromes, and coping styles, including social learning as a concept, to further elucidate pain‐processing in preterm‐born children and adolescents.

## 5 CONCLUSION

We observed a higher prevalence of increased sensitivity to pain in preterm‐born adolescents. EP adolescents were 2.5 times more likely to report increased sensitivity to pain compared with their peers, whereas MLP adolescents were 1.5 times more likely to report increased sensitivity to pain. The latter thus reported increased pain sensitivity more frequently than their FT peers, but less frequently than their EP peers. Increased sensitivity to pain of EP adolescents was associated with inotrope treatment during NICU stay. In contrast to self‐reported sensitivity to pain, parent‐reported sensitivity to pain did not differ by gestational age group. Our findings suggest that NICU stay and specific treatments could have consequences for the sensitivity to pain of preterm‐born adolescents.

## CONFLICTS OF INTEREST

The authors have no relevant conflicts of interest, financial or otherwise, to disclose.

## Supporting information

Table S1
