# Supplementary material for: Self‐reported sensitivity to pain in early and moderately‐late preterm‐born adolescents: A community‐based cohort study
Source: Paediatr Neonatal Pain. 2021 May 11;3(2):59–67. doi: 10.1002/pne2.12053 (PMC8975215; doi:10.1002/pne2.12053)
Supplement: Supplementary file 1 — Table S1 [file PNE2-3-59-s001.docx]

| **Supplementary Table 1.** Comparison in background characteristics between participants and non-participants in the adolescent follow-up wave of the LOLLIPOP study. | | | | | | |
| --- | --- | --- | --- | --- | --- | --- |
|  | | | **Participants (N=294)** | **Non-participants (N=537)** | **P-value** | **Effect size (Cohen’s W)** |
| Sex | | |  |  | 0.015 | 0.08 |
|  | Male | | 138 (46.9) | 299 (55.7) |  |  |
|  | Female | | 154 (52.4) | 234 (43.8) |  |  |
| Small-for-gestational age | | | 44 (15.0) | 50 (9.3) | 0.015 | 0.08 |
| Gestational age category | | |  |  | 0.002 | 0.12 |
|  | | Early preterm | 82 (27.9) | 92 (17.1) |  |  |
|  | | Moderately-late preterm | 130 (44.2) | 264 (49.2) |  |  |
|  | | Fullterm | 82 (27.9) | 181 (33.7) |  |  |
| Maternal educational level (N=807) | | |  |  | 0.005 | 0.10 |
|  | | Low/middle | 188 (63.9) | 387 (72.1) |  |  |
|  | | High | 100 (34.0) | 132 (24.6) |  |  |
| *Maternal educational level was measured upon inclusion in the LOLLIPOP study (age 4 years), and categorized as: low/middle educational level, i.e. <12 years of formal education and high educational level, i.e. ≥12 years of formal education.* | | | | | | |
